# Progestin effects on cell proliferation pathways in the postmenopausal mammary gland

**DOI:** 10.1186/bcr3456

**Published:** 2013-08-12

**Authors:** Charles E Wood, Daniel Branstetter, Allison P Jacob, J Mark Cline, Thomas C Register, Kathy Rohrbach, Li-Ya Huang, Hermina Borgerink, William C Dougall

**Affiliations:** 1 Department of Pathology, Section on Comparative Medicine, Wake Forest University School of Medicine, Winston-Salem, NC 27157, USA; 2 Department of Pathology, Amgen Inc, Seattle, WA 98119, USA; 3 Therapeutic Innovation Unit (TIU), Amgen Inc, Seattle, WA 98119, USA

**Keywords:** Postmenopausal hormone therapy, Estrogen, Progestin, Tibolone, Breast cancer, Gene expression, RANKL, Denosumab

## Abstract

**Introduction:**

Menopausal hormone therapies vary widely in their effects on breast cancer risk, and the mechanisms underlying these differences are unclear. The primary goals of this study were to characterize the mammary gland transcriptional profile of estrogen + progestin therapy in comparison with estrogen-alone or tibolone and investigate pathways of cell proliferation in a postmenopausal primate model.

**Methods:**

Ovariectomized female cynomolgus macaque monkeys were randomized into the following groups: placebo (Con), oral conjugated equine estrogens (CEE), CEE with medroxyprogesterone acetate (MPA) (CEE + MPA), and tibolone given at a low or high dose (Lo or Hi Tib). All study treatment doses represented human clinical dose equivalents and were administered in the diet over a period of 2 years.

**Results:**

Treatment with CEE + MPA had the greatest effect on global mRNA profiles and markers of mammary gland proliferation compared to CEE or tibolone treatment. Changes in the transcriptional patterns resulting from the addition of MPA to CEE were related to increased growth factors and decreased estrogen receptor (ER) signaling. Specific genes induced by CEE + MPA treatment included key members of prolactin receptor (PRLR)/signal transducer and activator of transcription 5 (STAT5), epidermal growth factor receptor (EGFR), and receptor activator of nuclear factor kappa B (RANK)/receptor activator of nuclear factor kappa B ligand (RANKL) pathways that were highly associated with breast tissue proliferation. In contrast, tibolone did not affect breast tissue proliferation but did elicit a mixed pattern of ER agonist activity.

**Conclusion:**

Our findings indicate that estrogen + progestin therapy results in a distinct molecular profile compared to estrogen-alone or tibolone therapy, including upregulation of key growth factor targets associated with mammary carcinogenesis in mouse models. These changes may contribute to the promotional effects of estrogen + progestin therapy on breast cancer risk.

## Introduction

Different types of postmenopausal hormone therapy (HT) have been widely used for more than 60 years to alleviate symptoms of menopause and prevent associated conditions such as osteoporosis. The primary form of HT during much of this time has been estrogen-alone therapy (ET) [1]. In the mid-1990s, the clinical use of estrogen plus progestin therapy (EPT) began after several studies demonstrated that progestins opposed the adverse effects of ET on endometrial cancer risk [2]. These findings helped spur a rapid increase in EPT prescriptions from less than two million in 1995 to 24 million in 2001 [1]. At the time, the most common type of HT used in the US was conjugated equine estrogens (CEE) with or without the progestin medroxyprogesterone acetate (MPA), which together accounted for more than 60% of total HT prescriptions [1].

Use of HT fell dramatically in 2002 after the release of the primary results from the Women’s Health Initiative (WHI) Estrogen + Progestin Trial [1,3]. In this large randomized clinical trial, postmenopausal women receiving EPT (CEE + MPA) treatment had significantly higher incidence of invasive breast cancer compared with those taking the placebo [3,4]. Subsequent reports noted increased breast cancer mortality for women taking EPT [5] and decreased breast cancer incidence following discontinuation of EPT [6]. These results supported prior epidemiologic studies [7,8] but differed from the sister WHI Estrogen-Alone Trial, in which CEE alone did not increase the incidence of invasive breast cancer among women with a prior hysterectomy [9,10]. Collectively, these studies confirmed that the promotional effects of EPT on certain types of breast cancer were greater than those seen with estrogen alone.

A prominent alternative to traditional menopausal HTs used outside the US is tibolone. This unique steroidal compound is converted in a tissue-selective manner to estrogenic, progestogenic, and androgenic metabolites. Estrogenic metabolites have been shown to reduce menopausal symptoms and fracture risk [11,12], whereas the progestogenic Δ-4 isomer metabolite (selectively produced in the uterus) has been shown to provide endometrial protection from estrogenic effects [13,14]. However, the effects of tibolone therapy on breast cancer risk are controversial. In a randomized clinical trial of older postmenopausal women, tibolone therapy resulted in a significantly lower breast cancer risk [12], whereas in a separate clinical trial, breast cancer survivors had increased risk of recurrence following tibolone treatment [15].

Despite numerous preclinical and clinical studies, the mechanistic actions of different HTs on breast tissue have not been clearly defined. In mouse models, ovarian hormones contribute significantly to mammary gland carcinogenesis [16]; however, differences between mouse and human mammary gland anatomy, development, and/or hormonal control of proliferation may limit the translational relevance of targets identified in the mouse for estrogen and progestogen function. Macaques are anthropoid primates with high overall genetic coding sequence identity to humans, including key genes related to breast cancer susceptibility [17]. Prior work has shown close similarities between macaque and human mammary gland biology, including responses to exogenous estrogen and progestogen therapies, sex steroid receptor expression, and age-related hyperplastic and neoplastic lesions [18]. This includes a study on aged rhesus macaques and suggests a lifetime incidence of mammary gland cancer at about 6% [19], similar to lower-risk human populations. Previous studies in this model have also shown that the addition of a progestin to an estrogen increases mammary gland proliferation and density beyond that seen with estrogen alone, in support of the later WHI findings related to breast cancer risk [20]. The primary aim of this study was to assess the effects of long-term treatment with CEE, CEE + MPA, or tibolone on the transcriptional profiles and signaling pathways of the normal postmenopausal primate mammary gland. Our main goals were to identify specific targets associated with hormone-induced breast proliferation and evaluate their relations with candidate pathways known to drive mammary carcinogenesis in mouse tumor models.

## Methods

### Study design and treatments

This study utilized archived tissue samples from a parallel-arm design experiment in which 149 ovariectomized adult female cynomolgus macaques (*Macaca fascicularis*) with a mean estimated age of six to eight years were randomized to receive one of the following five treatments for two years: placebo (control; n = 31); CEE at 0.042 mg/kg (CEE; n = 28); CEE + MPA at 0.167 mg/kg (CEE + MPA; n = 29); tibolone at 0.05 mg/kg (Lo Tib; n = 30); and tibolone at 0.2 mg/kg (Hi Tib; n = 31). Dose equivalents approximated standard HT doses of CEE (0.625 mg/day) and MPA (2.5 mg/day) in postmenopausal women, and tibolone doses were designed to approximate 0.75 mg/day (low) and 3.0 mg/day (high) doses in women. Serum concentrations of estrogens, MPA, and tibolone metabolites were similar to those reported in women taking comparable therapies [21]. Treatments were administered in the control diet with casein plus lactalbumin as the protein source and macronutrient composition based on a typical human diet in the USA [22]. Animals were housed in social groups of five animals each and fed 60 kcal/kg (plus 10% extra to account for waste) twice daily, with drug treatments split between the two feedings. Daily doses were scaled to 1,800 kcal of diet (the estimated daily intake for a US woman) to account for differences in metabolic rates between monkeys and human subjects. All animals were considered multiparous based on historical data from the original breeding colony and uterine histology. Histology outcomes were described previously [21]; no mammary gland tumors were detected.

All procedures involving macaques in this study were conducted in compliance with State and Federal laws and standards of the US Department of Health and Human Services and were approved by the Wake Forest University Animal Care and Use Committee. The facilities and laboratory animal program of Wake Forest University are fully accredited by the Association for the Assessment and Accreditation of Laboratory Animal Care.

### Gene microarray analyses

Mammary gland tissues were collected during necropsy at the end of the two-year treatment period [21], and designated portions were snap-frozen in liquid nitrogen and stored at −70°C for gene expression analyses. Total mammary gland RNA was extracted from frozen samples using Tri Reagent (Molecular Research Center, Cincinnati, OH, USA), purified using RNeasy Mini kit (QIAGEN, Valencia, CA, USA), and quantitated using a NanoDrop ND-1000 UV–vis spectrophotometer (NanoDrop, Wilmington, DE, USA). Nucleic acid intactness and quality were confirmed using an Agilent 2100 Bioanalyzer (Agilent Technologies, Wilmington, DE, USA). Biotinylated cRNA samples were prepared according to the standard Enzo Bioarray™ protocol (Enzo Life Sciences, Farmingdale, NY, USA) and hybridized using the standard Affymetrix (Santa Clara, CA, USA) protocol for eukaryotic samples. Biotinylated cRNA from each sample was hybridized to Affymetrix GeneChip Rhesus Macaque Genome Arrays (Santa Clara, CA, USA), washed and stained in an Affymetrix GeneChip Fluidics Station (Santa Clara, CA, USA), and scanned with an Affymetrix GeneChip® Scanner 3000 (Santa Clara, CA, USA). Intensity data were extracted from scanned images and checked for quality using Affymetrix GeneChip Operating Software and Expression Console (MAS5 algorithm; Santa Clara, CA, USA). Microarray assays were performed at Beckman Coulter Genomics (Morrisville, NC, USA). Four randomly selected samples per group were run on microarray from control, CEE, CEE + MPA, and Hi Tib groups.

Microarray data analyses were performed using the GeneSifter® software program (Perkin Elmer/Geospiza, Seattle, WA, USA). Intensity data were RMA-normalized, converted to a log_2_ scale, screened for heterogeneity among samples and groups, and evaluated using supervised analysis of variance (ANOVA) and pairwise comparisons between treatments. Principal components analysis (PCA), pattern navigation, cluster analysis, heat mapping, and KEGG pathway analyses were performed on filtered data subsets, as described in the Results section. Differences in gene numbers altered by each treatment were compared using either Fisher’s exact test or chi-square test. Euclidean distances (representing the numeric difference between treatment vectors) were calculated as part of hierarchical clustering dendrograms using average linkage. Pathways related to cell proliferation were evaluated using *z*-scores generated in KEGG analyses; a *z*-score more than 2.0 was considered significant overrepresentation of genes in a particular pathway. All *P* values were corrected when possible for multiple comparisons using the Benjamini and Hochberg method (*P*_*adj*_), which derives a false discovery rate estimate from the raw *P*-values [23]. Representation of differentially expressed genes within specific functional categories was evaluated using Ingenuity Pathway Analysis software v6 (Ingenuity Systems, Redwood City, CA, USA) using a Fisher’s exact test with Benjamini and Hochberg correction and expressed as -log_10_ of the *P* value for gene numbers within each treatment group. Microarray data are publicly available on the NCBI Gene Expression Omnibus database (accession number GSE27228).

### Quantitative gene expression

Expression of genes associated with proliferation, epithelial density, growth factor signaling, oestrogen receptor (ER) expression and activity, estrogen metabolism, and receptor activator of nuclear factor kappa-B (RANK)/RANK ligand (RANKL) pathway activity were measured in mammary gland samples using quantitative real-time reverse transcriptase polymerase chain reaction (qPCR). Macaque-specific qPCR primer-probe sets for internal control genes (glyceraldehyde-3-phosphate dehydrogenase *(GAPDH)*; beta-actin *(ACTB*)) were generated through the Applied Biosystems Taqman Assay-by-Design service (Foster City, CA, USA). Sources for all target primer/probe sets are given in Additional file 1: Table S1. All probes spanned an exon-exon junction to eliminate genomic DNA amplification. qPCR reactions (20 μl volume) were performed on an Applied Biosystems 7500 Fast Real-Time PCR system (Foster City, CA, USA) using standard Taqman reagents and thermocycling protocol [24]. Relative expression was determined using the ΔΔCt method. The Ct values for the control genes *GAPDH* and *ACTB* were averaged for use in internal calibration, and premenopausal breast tissue RNA was a reference for plate-to-plate parallel calibration. Calculations were performed using Applied Biosystems Relative Quantification 7500 Software v2.0.1 (Foster City, CA, USA).

### Immunohistochemistry

Immunohistochemical (IHC) staining was performed on fixed paraffin-embedded mammary gland sections. Slides were deparaffinized, rehydrated in water, prepared by heat-induced epitope retrieval using Diva Decloaker (Biocare Medical, Concord, CA, USA) and Decloaking Chamber Plus (Biocare Medical, Concord, CA, USA) at heat and pressure cycles of 125°C for 30 and 10 seconds. Slides were gradually cooled by replacing the retrieval solution with deionized water and rinsed twice in wash buffer (Dako Wash Buffer, DakoCytomation Carpinteria, CA; five minutes) before loading on a Dako Autostainer (Dako North America Inc, Carpinteria, CA). Sections were blocked for endogenous peroxidases and nonspecific binding of staining reagents by sequentially incubating with 3% hydrogen peroxidase (hydrogen peroxidase block, Thermo Scientific Waltham, MA; 10 minutes), Avidin (Vector Labs, Burlingame, CA; 15 minutes), Biotin (Vector Labs, Burlingame, CA; 15 minutes), and TNB (Perkin Elmer; 20 minutes). Tris-NaCl-blocking buffer was removed and replaced with anti-human RANK (N-2B10.1 or N-1H8.1; Amgen, Seattle, WA) or RANKL (M366; Amgen, Seattle, WA) mouse monoclonal antibodies or isotype-matched control mouse IgG (BD Pharmingen, San Jose, CA) at concentrations of 5 μg/mL for anti-RANK and 1 μg/mL for anti-RANKL for 60 minutes. A biotinylated, goat anti-mouse IgG (Vector Labs, Burlingame, CA) secondary antibody in 10% normal human serum Tris-NaCl-blocking buffer was applied at a concentration of 7.5 μg/mL followed by a 30 minute incubation. Slides were sequentially incubated with streptavidin-horseradish peroxidase (SA-HRP; Perkin Elmer, Waltham, MA; 30 minutes) at a 1:1500 dilution in TNB, tyramide signal amplification TSA (Perkin Elmer, Waltham, MA; five minutes) at a 1:100 dilution in amplification diluent (Perkin Elmer, Waltham, MA), and then SA-HRP (Perkin Elmer, Waltham, MA; 30 minutes) at a 1:1500 dilution in TNB. Slides were then incubated with diaminobenzidine chromogen (Dako, Carpinteria, CA; five minutes), counterstained with hematoxylin (Dako, Carpinteria, CA; 30 seconds), allowed to turn blue in tap water for two minutes before dehydrating with ascending concentrations of ethanol, cleared with xylene, and mounted.

The intensity of IHC staining was scored on a semiquantitative scale (0 = absent, 1 = weak, 2 = moderate, 3 = intense), blinded to treatment group by a board-certified pathologist. Incidence was scored as a positive IHC signal (any intensity). Immunostaining of slides for Ki-67 antigen was described previously [21]. For dual labeling experiments, the following modifications to the above procedure were performed. Antigen retrieval was performed using Diva AR reagent (Biocare, Concord, CA; DV2004G1) at 90°C overnight in the Decloaking Chamber. Sections were blocked as described above, incubated with either anti-progesterone receptor PGR (Dako, Carpinteria, CA; M3569 at 1:40; 40 minutes) or anti-Ki-67 (Epitomics, Burlingame, CA; 4203–1 at 1:400; 60 minutes), detected with Dako Mouse or Rabbit Envision + Systems (Dako, Carpinteria, CA, 30 minutes), and visualized by Dako DAB+ (Dako, Carpinteria, CA, 10 minutes). Antibody staining from the first PR/Ki-67 IHC incubation/staining were blocked by rinsing sections in distilled water, eluting, and incubating the slides in Diva AR reagent (Biocare, Concord, CA) at 98°C for 10 minutes. Slides were washed and blocked as described above; followed by incubation with either anti-RANKL (M366 at 2 μg/mL), anti-RANK (N-1H8.1 at 5 μg/mL), or mouse IgG1 isotype control (5 μg/mL) for 60 minutes. Secondary antibody incubation was performed as described above, followed by incubation in streptavidin alkaline phosphatase, tyramide amplification and repeat of strepavidin alkaline phosphatase. Slides were then incubated with permanent red chromogen (Dako, Carpinteria, CA; 20 minutes), counterstained with Mayer’s hematoxylin, washed and aqueous mounted. Histologic images were photographed using a Nikon Eclipse E600 microscope with a Nikon (Tokyo, Japan) DXM1200 digital camera. The resulting images were white-balanced using Adobe Photoshop CS software (San Jose, CA); no additional image modifications were employed.

### Statistical analyses

Data were analyzed using the SAS statistical package v9 (SAS Institute, Cary, NC, USA). All data were evaluated for normal distribution and homogeneity of variances among groups. Gene expression data were evaluated using a nonparametric Kruskal-Wallis test followed by two-sided Wilcoxon rank sum pairwise analysis and reported as a fold-change of control with 90% confidence interval. One control group animal died during the course of the study, reducing the number of control animals to 30. All pairwise *P* values were adjusted for the number of pairwise tests using a Bonferroni correction. Pre-planned pairwise tests included each treatment group versus control and CEE + MPA versus CEE and Hi Tib groups. Intensity of RANK/RANKL immunostaining was evaluated using a one-way ANOVA with Bonferroni’s post test. To evaluate the correlation of gene or protein expression with IHC, data were log transformed and linear regression analysis was performed. A two-tailed significance level of 0.05 was used for all comparisons.

## Results

### EPT elicits distinct effects on global gene expression profiles

**Figure 1:**
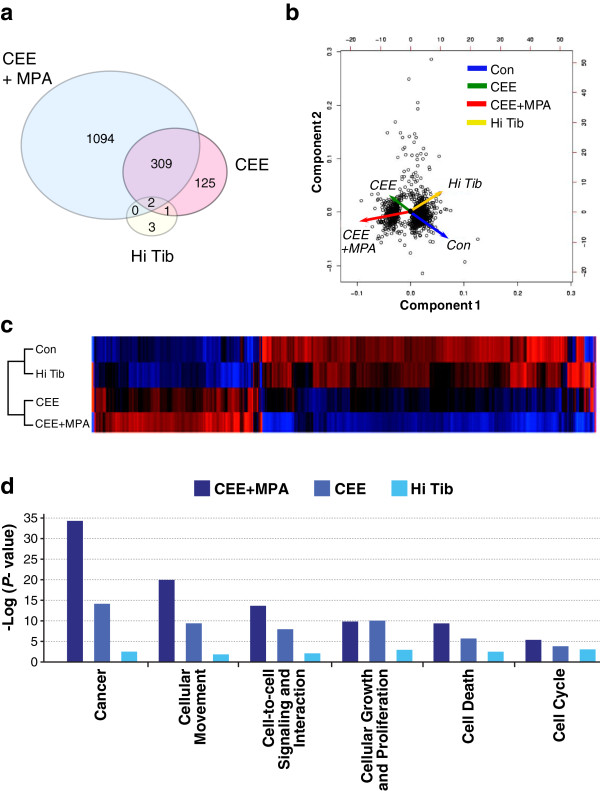
**Global transcriptional patterns in the breast tissue following treatment with control (placebo), conjugated equine estrogens (CEE), CEE + medroxyprogesterone acetate (MPA), and high-dose tibolone (Hi Tib). (a)** Venn diagram showing greater numbers of genes altered by CEE + MPA compared with CEE and Hi Tib. **(b)** principal component analysis and **(c)** heatmap of differentially regulated genes showing greater overlap between Con and Hi Tib groups and the CEE + MPA and CEE groups. **(d)** Functional categories of significantly altered transcripts showing overrepresentation of genes involved in cancer and several related classes. mRNA profiles were determined by microarray analysis. All diagrams correspond to significantly altered transcripts with fold-change of more than 1.5 versus control group in at least one group, adjusted analysis of variance (ANOVA) *P* < 0.05, and quality more than 2.

Global mammary gland expression profiles were evaluated by microarray analysis. A total of 52,865 array probe sets were detected at a quality score of more than 2.0. Of these, probes for 1,534 different genes were significantly altered at a fold change (FC) more than 1.5 and an adjusted ANOVA *P* < 0.05. HTs resulted in distinct effects on mammary gland gene expression. Overall transcriptional effects were greatest for CEE + MPA and lowest for Hi Tib. For example, CEE + MPA resulted in a greater number of significantly altered genes versus controls (n = 1405) compared with CEE (n = 437) and Hi Tib (n = 6) (*P* < 0.0001) (Figure 1a). Among these genes, PCA and heatmap analysis showed modest overlap in transcriptional profiles for CEE and CEE + MPA and a profile for Hi Tib most similar to the control group (Figure 1b, 1c). Functional analysis of significantly altered genes showed overrepresentation of several categories related to cell proliferation and cancer risk (Figure 1d). The most highly represented functional gene category was cancer. Within this class, a greater number of differentially regulated genes was seen for CEE + MPA (n = 497, *P* < 10^-34^) compared with CEE (n = 166, *P* = 10^-14^) and Hi Tib (n = 2, *P* = 0.01). Other functional categories showing greater overrepresentation in the CEE + MPA group included cellular movement, signaling, and interaction (Figure 1d).

### EPT increases cell proliferation and growth factor signaling markers

**Figure 2:**
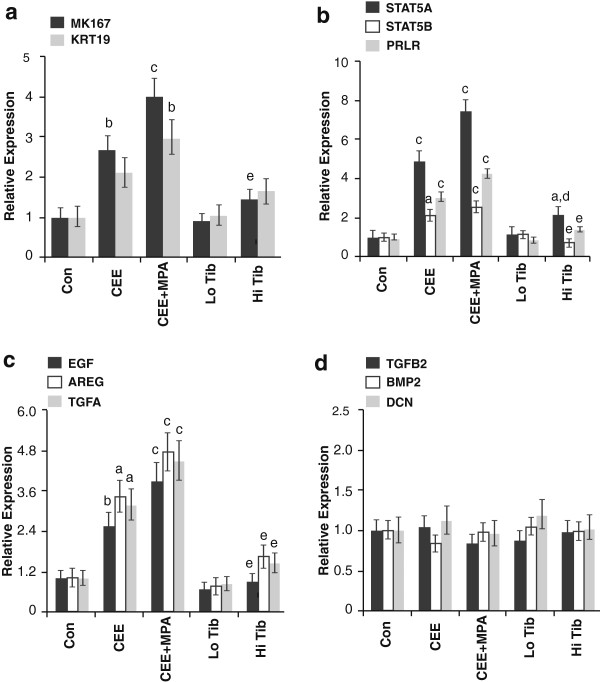
**Hormone therapy effects on gene markers related to mammary gland proliferation. (a)** markers included cell proliferation (*MKI67*) and epithelial density (*KRT19*) and genes involved in the following signaling pathways; **(b)** STAT5; **(c)** EGFR; and **(d)** TGF-beta. mRNA levels were determined by quantitative PCR. Vertical lines indicate 90% confidence interval. Letters indicate significant differences compared with control (*P* < 0.05^*a*^, *P* < 0.01^*b*^, *P* < 0.001^*c*^) and conjugated equine estrogens plus medroxyprogesterone acetate (CEE + MPA), (*P* < 0.01^*d*^, *P* < 0.001^*e*^) groups.

Similar to global profiles, quantitative expression of specific markers for epithelial proliferation (antigen identified by monoclonal antibody Ki-67 (*MKI67*)) and density (keratin 19 (*KRT19*)) was highest for CEE + MPA, intermediate for CEE, and lowest for tibolone groups compared with placebo (Figure 2a). Treatment effects related to cell proliferation included the Jak/Stat and ErbB pathways, which were overrepresented among upregulated genes, and the transforming growth factor (TGF)-beta pathway, which was significantly overrepresented among downregulated genes. On microarray analysis, Jak/Stat and ErbB-related genes related primarily to signal transducer and activator of transcription 5 (STAT5) and epidermal growth factor receptor (EGFR) signaling pathways, respectively. On qPCR, expression of key molecules within these pathways was highest for CEE + MPA treatment, showing similar overall expression patterns to that seen for *MKI67* (Figure 2b, 2c) and high correlation for individual markers, particularly *STAT5A* and amphiregulin (*AREG*) (*R* > 0.7, *P* < 10^-20^ for both) [see Additional file 2: Figures S1a, S1b]. In contrast, no significant treatment effects were seen on qPCR for individual markers of TGF-beta pathway activity (Figure 2d).

### Adding a progestin to ET inhibits ER activity

**Figure 3:**
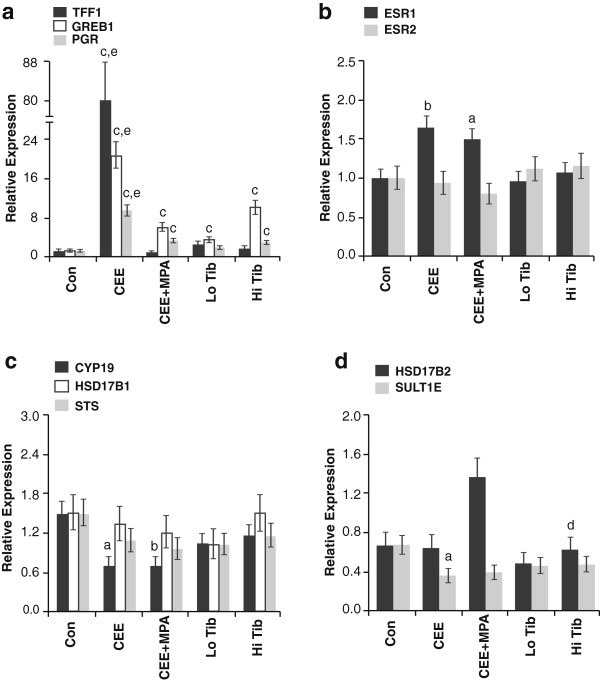
**Hormone therapy effects on gene markers related to ER expression and activation. (a)** Hormone therapy effects on gene markers related to oestrogen receptor (ER) activation; **(b)** ER expression; **(c)** estradiol synthesis and bioactivation; and **(d)** estradiol deactivation. mRNA expression levels were determined by quantitative PCR. Vertical lines indicate 90% confidence interval. Letters indicate significant differences compared with control (*P* < 0.05^*a*^, *P* < 0.01^*b*^, *P* < 0.001^*c*^) and conjugated equine estrogens plus medroxyprogesterone acetate (CEE + MPA) (*P* < 0.01^*d*^, *P* < 0.001^*e*^) groups.

We next examined whether patterns of ER activity were associated with treatment differences in proliferation and growth factor expression. Treatment with CEE markedly induced gene markers of ER activity, whereas the addition of MPA completely or partially antagonized this effect (depending on the marker). For example, CEE increased trefoil factor 1 (*TFF1*) expression by 82-fold, whereas CEE + MPA was not different from placebo (Figure 3a). For other ER-induced markers such as progesterone receptor (*PGR*) and growth regulation by estrogen in breast cancer 1 (*GREB1*), the addition of MPA blocked 75% and 71% of CEE-induced expression, respectively. This pattern was also present on microarray analysis, where the primary cluster of genes differentially altered between CEE and CEE + MPA groups were related to ER signaling. The gene cluster included *TFF1*, *PGR*, *GREB1*, and other ER-sensitive genes such as insulin-like growth factor binding protein 1 (*IGFBP1*), breast carcinoma amplified sequence, and fibulin. Tibolone had a mixed pattern of effects on ER activity, inducing *PGR* and *GREB1* (*P* < 0.001 for both compared with control) but not *TFF1* at the higher dose (Figure 3a). Treatment effects on ER activity were not directly associated with changes in expression of ER-alpha (*ESR1*), ER-beta (*ESR2*) (Figure 3b), or key genes related to estradiol metabolism (Figure 3c, 3d). However, ER immunolabeling was lower for *ESR1* following CEE + MPA but not CEE as described previously [25]. In contrast to growth factors, markers of ER activity showed modest (*PGR* and *GREB1*) or no (*TFF1*) significant correlation with proliferation [see Additional file 2: Figure S1c].

### Tibolone treatment does not induce growth factor signals

The main transcriptional pattern among genes altered by the Hi Tib dose related to ER signaling; no other clear patterns were noted. Of the 24 identified genes with more than three FC and *P* < 0.05 compared with control, 20 genes were upregulated. Among these, there were seven known ER-sensitive genes (*PGR*, *GREB1*, stanniocalcin 2 *[STC2]*, secretoglobin family 1D member 2 *[SCGB1D2]*, kallikreins 11 *[KLK11]* and 12 *[KLK12]*, and *IGFBP1*); two genes involved in steroid metabolism (cytochrome p450 family 2 subfamily A polypeptide 7 *[CYP2A7]* and 3-beta-hydroxysteroid dehydrogenase ∆-5-∆-4 isomerase type II *[HSD3B2]*); two secretoglobins *(SCGB3A1* and *SCGB1D2)*; and three peptidases (disintegrin and metalloproteinase with thrombospondin motifs 8 *[ADAMTS8]*, *KLK11*, and *KLK12*). Notably, isoforms of HSD3B are the primary enzymes driving production of the progestogenic delta-4 tibolone metabolite [13]. Despite a 6.5 FC increase in *HSD3B2* expression in the Hi Tib group, no markers of progestogenic activity were noted.

### EPT increases RANK/RANKL pathway expression

**Figure 4:**
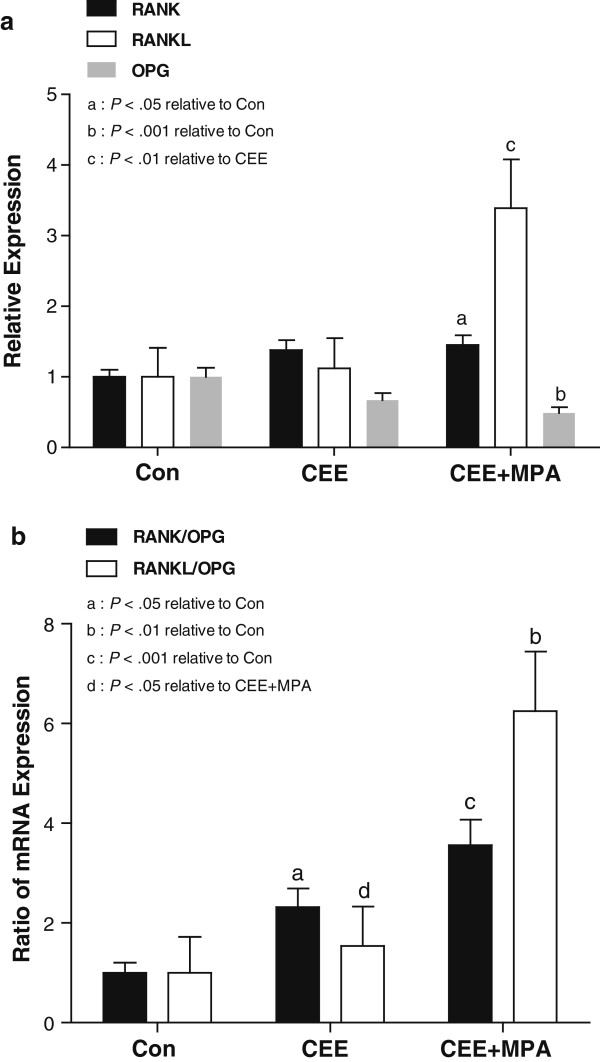
**Hormone therapy effects on gene markers of RANK/RANKL signaling. (a)** mRNA expression of *RANK*, *RANKL*, and the endogenous inhibitor of RANKL signaling (*OPG*) and **(b)** ratios between these markers. mRNA levels were determined by quantitative PCR and expressed as **(a)** relative means or as **(b)** ratios of *RANK*/*OPG* or *RANKL*/*OPG*. Vertical lines indicate 90% confidence interval. Letters indicate significant differences between groups as indicated.

**Figure 5:**
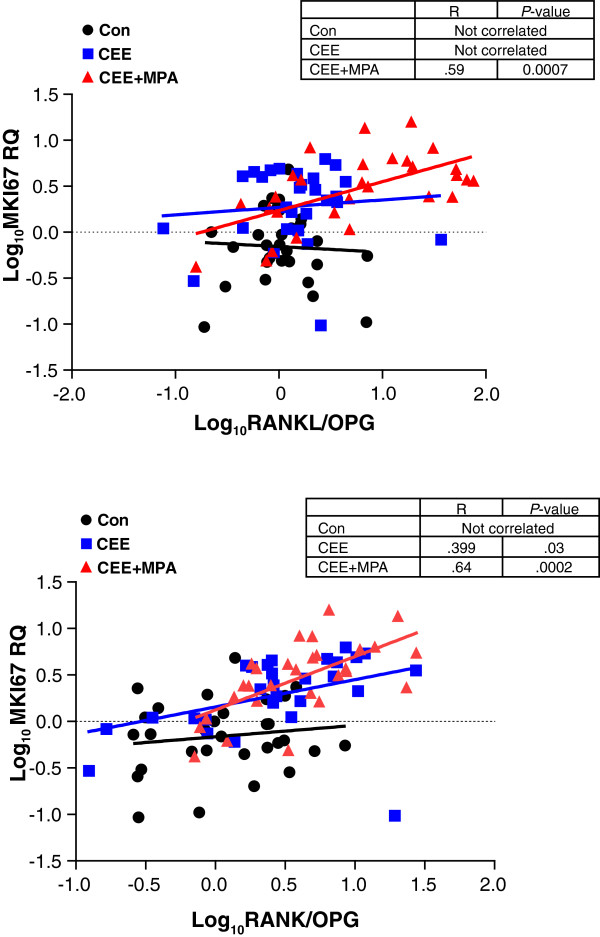
**Regression analysis for expression of the proliferation marker *MKI67 *versus *RANKL*/*OPG *or *RANK*/*OPG *mRNA ratios.** Correlation of *MKI67* and the *RANKL*/*OPG* mRNA ratio was only observed within the conjugated equine estrogens plus medroxyprogesterone acetate (CEE + MPA) group and not in the control or CEE groups. The strongest correlation of *MKI67* and *RANK*/*OPG* mRNA ratio was observed in the CEE + MPA group.

The RANK pathway exhibits crosstalk with both STAT5 [26] and EGFR [27] signaling and contributes to progestogen-induced cell proliferation in the mouse mammary gland [28,29]. Mouse mammary expression of RANKL is increased substantially after progestin treatment [30], and increased RANK expression is observed at ductal side branches and alveoli during pregnancy-induced mammary morphogenesis [31]. To further explore hormonal regulation of this pathway in the primate mammary gland, we evaluated ET and EPT effects on expression of mRNAs encoding *RANK*, *RANKL*, and the endogenous inhibitor of RANKL, osteoprotegerin (*OPG*). No significant group differences in *RANK*, *RANKL*, or *OPG* expression were noted in analysis by microarray. By qPCR, treatment with CEE alone did not result in significant changes in *RANK*, *RANKL,* or *OPG* expression (Figure 4a), whereas treatment with CEE + MPA resulted in a significantly higher expression of *RANK* and lower expression of *OPG* relative to control (*P* < 0.05 and *P* < 0.001, respectively; Figure 4a). Treatment with CEE + MPA also increased *RANKL* mRNA more than three-fold versus control and the differences between *RANKL* mRNA levels after CEE + MPA versus CEE treatment were statistically significant (*P* < 0.01). Analysis of *RANKL* mRNA changes was confounded by one animal in the CEE-treated cohort which had more than 50-fold higher levels of *RANKL* mRNA than all other animals. Treatment with CEE + MPA resulted in greater ratios of *RANK* and *RANKL* to *OPG* compared with control (*P* < 0.001 and *P* < 0.01, respectively; Figure 4b) and greater ratio of *RANKL* to *OPG* compared with the CEE group (*P* < 0.05; Figure 4b). Although *RANKL* mRNA levels did not correlate with *MKI67* [see Additional file 3: Figure S2a], the ratios of *RANKL*:*OPG* and *RANK*:*OPG* expression showed a significant positive association with *MKI67* (Figure 5), with the strongest correlations observed in the CEE + MPA group (R = 0.59, *P* = 0.0007 and R = 0.64, *P* = 0.0002, respectively). The *RANK*/*OPG* ratio expression also showed a significant positive association with *STAT5A* (R = 0.69, *P* < 0.0001) and *KRT19* (R = 0.55, *P* < 0.0001), whereas *RANKL*/*OPG* ratio showed significant positive, yet modest, correlations with *STAT5A* and *KRT19* (R = 0.35, *P* = 0.0008 and R = 0.29, *P* = 0.006, respectively) [see Additional file 4: Figure S3].

### RANKL and RANK protein show distinct patterns of expression in the mammary gland

**Figure 6:**
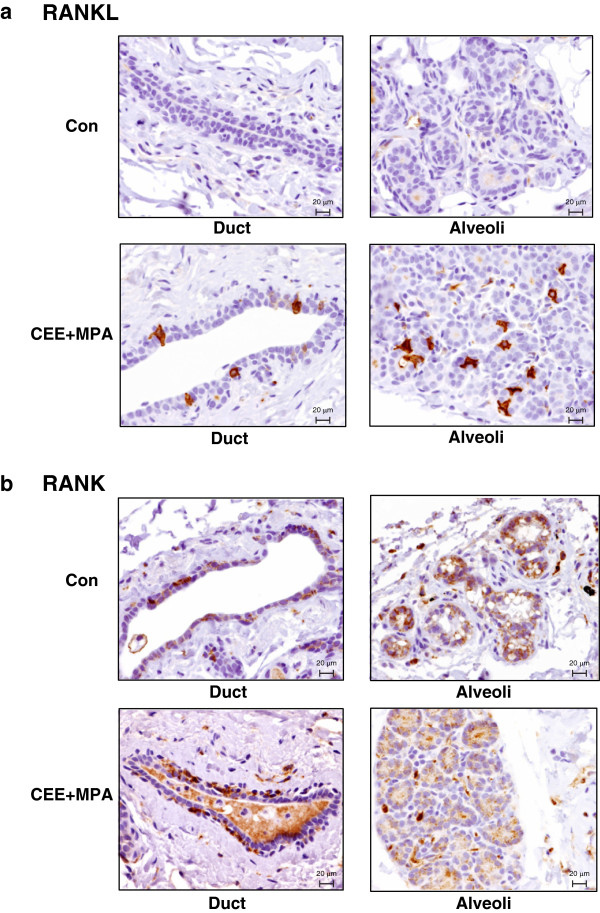
**Expression and localization of RANKL and RANK protein within the mammary gland. (a**,**b)** Representative images of RANKL and RANK immunohistochemical staining in control and conjugated equine estrogens plus medroxyprogesterone acetate (CEE + MPA) groups. There is no detectable staining for RANKL in the control group but RANKL protein is clearly evident exclusively within the luminal epithelial cells of the lobuloalveolar and ductal epithelium in the CEE + MPA group. RANKL was not detected within myoepithelial, stromal, or infiltrating immune cells. RANK expression was observed in both ductal and lobuloalveolar structures in all groups. RANK protein was predominately observed in basal cells in close proximity to the basement membrane and also in some epithelial cells, which extended from the basement matrix/basal layer to the lumen. The observed staining of intraluminal secretory material using the RANK antibodies was due to non-specific binding to proteinaceous material in serum (data not shown).

**Figure 7:**
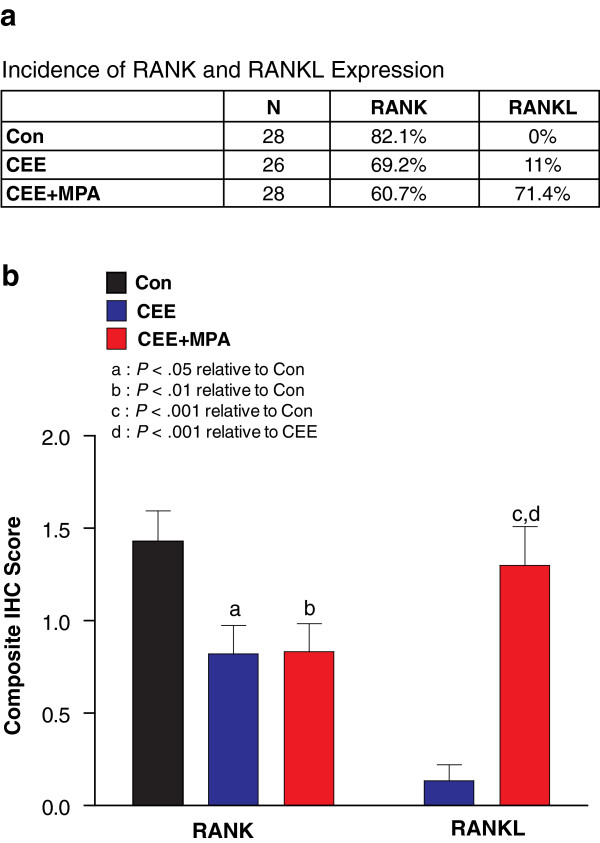
**Hormone therapy effects on incidence of expression and intensity of IHC staining for RANK and RANKL. (a)** Incidence score for RANK or RANKL immunohistochemical (IHC) positivity in each group. **(b)** Mean composite IHC score +/− standard error of the mean in each treatment group. Letters indicate significant differences versus control (*P* < 0.05^*a*^, *P* < 0.01^*b*^, *P < 0*.001^*c*^) or conjugated equine estrogens (CEE) (*P* < 0.001^*d*^) groups.

We next utilized IHC with monoclonal antibodies against huRANKL or huRANK to verify that the observed changes in mRNA expression were translated into protein and to determine precise cellular localization for each protein. RANKL protein was not observed within the mammary epithelium of postmenopausal monkeys in the control group but was selectively increased in the CEE + MPA group (Figures 6a; 7a, b). In contrast, RANK protein in the mammary gland was prevalent across all groups (>50% of monkeys in each group; Figures 6b; 7a) with a modestly lower composite score for the CEE and CEE + MPA groups compared with control (*P* < 0.05 and *P* < 0.01, respectively; Figure 7b). RANKL protein expression was observed exclusively within luminal epithelial cells of ducts and lobuloalveolar structures, such that RANKL-positive cells were adjacent to RANKL-negative cells (Figure 6a). Dual immunostaining of samples from CEE + MPA-treated monkeys indicated that RANKL protein was localized in PGR-expressing luminal epithelial cells of ducts and lobuloalveolar structures [see Additional file 5: Figure S4], similar to what has been described in mice [28] and humans [32]. Expression of RANK protein was not uniform within the mammary gland, showing segmental foci of positive staining predominately within ducts and lobuloalveoli (Figure 6b). Furthermore, RANK protein expression was observed in basal cells and other epithelial cells that extended from the basal compartment to the lumen. Immunostaining of both RANK and RANKL was predominately limited to mammary epithelium, with rare expression in infiltrating cells (presumed lymphocytes). Staining was cytoplasmic and membranous for both RANK and RANKL, often with a granular cytoplasmic appearance for RANKL. This cellular distribution of RANK and RANKL protein within the monkey mammary gland was similar to that observed in mice [28,31] and tissue from normal human breast (Branstetter and Dougall, manuscript in preparation).

### RANKL and RANK protein expression is associated with mammary epithelial cell proliferation

**Figure 8:**
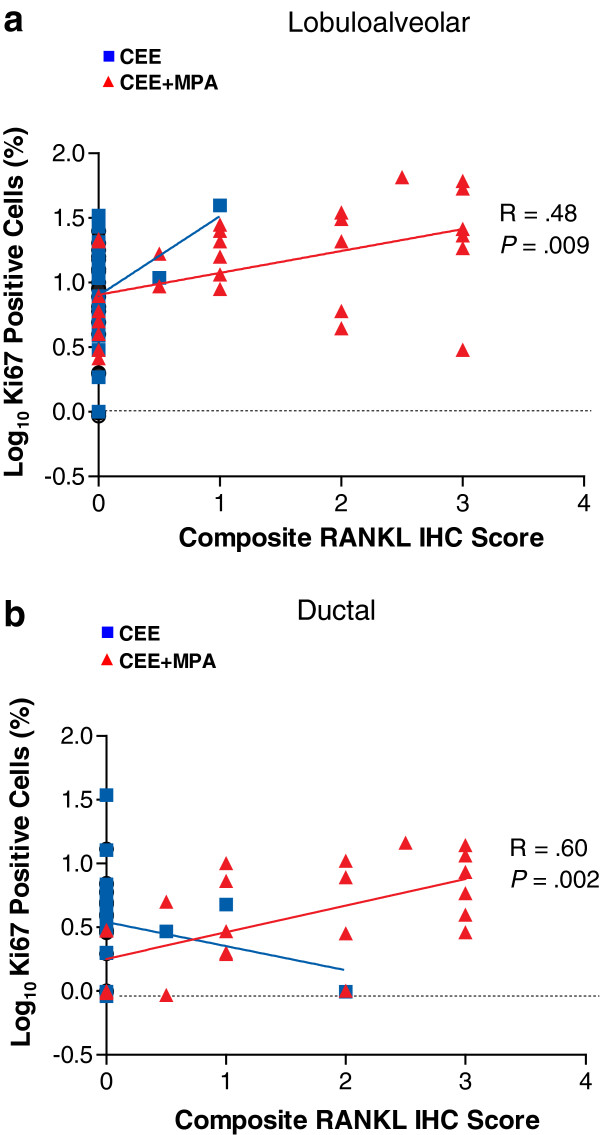
**Correlation of RANKL protein expression with Ki-67 protein expression.** Regression analysis for expression of the proliferation marker Ki-67 (immunohistochemical (IHC)) versus RANKL protein (IHC) in **(a)** lobuloalveolar or **(b)** ductal cells. Correlation between RANKL IHC score and percent Ki-67 positive cells is observed in conjugated equine estrogens plus medroxyprogesterone acetate (CEE + MPA) treated animals (R = 0.48, *P* = 0.009 for lobuloalveolar epithelia, R = 0.6, *P* = 0.002 for ductal epithelia). The majority of animals within the control and CEE groups (100% and 88%, respectively) did not express RANKL by IHC, precluding an accurate correlation analysis between RANKL and Ki-67 in these groups.

The intensity of RANKL protein expression determined by IHC showed a significant positive correlation with RANKL mRNA within the CEE + MPA group (R = 0.62, *P* = 0.0004) but not the control and CEE groups [see Additional file 6: Figure S5]. Previous analysis using Ki-67 IHC defined a mammary epithelial proliferative response specifically in the CEE + MPA group, with the majority of labeling in the lobuloalveolar compartment and minimal Ki-67 increases observed in large ducts [21]. Here, RANKL protein expression within the CEE + MPA group was significantly correlated with the degree of proliferation as determined by Ki-67 IHC in both alveoli (R = 0.48, *P* = 0.009; Figure 8a) and ducts (R = 0.6, *P* = 0.002; Figure 8b); there were no significant positive correlations of RANKL protein and Ki-67 IHC within control or CEE-treated groups (Figure 8a, 8b). RANK protein and mRNA were not significantly correlated in any group [see Additional file 6: Figure S5]. Although the intensity of RANK protein was not correlated with the degree of proliferation in any group [see Additional file 7: Figure S6], dual labeling of RANK and Ki-67 was observed in a subset of proliferating breast epithelial cells from CEE + MPA-treated monkeys. Segmental foci of breast epithelium that stained positively for RANK were also frequently positive for Ki-67 whereas RANK-negative regions of the same breast tissue often had few or no Ki-67 labeled cells [see Additional file 8: Figure S7]. In addition, clear examples of individual cells positive for both RANK and Ki-67 were observed in ducts and lobuloalveolar structures [see Additional file 8: Figure S7].

## Discussion

The addition of a progestin to ET is associated with increased breast tissue proliferation [33], mammographic density [4], and breast cancer risk in postmenopausal women [4,7,8]. Molecular mechanisms driving these effects are not clearly defined. In this study we show that adding the progestin MPA to CEE dramatically altered the mRNA profile in the normal primate mammary gland. This change was associated with greater mammary gland proliferation, decreased markers of ER activity, and increased markers of growth factor signaling. Many of the progestin-dependent changes observed here are also seen in the mouse mammary gland and have been associated with mammary carcinogenesis. These findings identify key differences among common types of menopausal HTs and highlight specific pathways relevant to hormonal promotion of mammary epithelial cell growth.

Our results show clear differences between ET and EPT effects on breast tissue and support the hypothesis that EPT increases cell proliferation beyond that of ET alone due in part to specific growth factor signals. Three primary progestin-regulated pathways were identified in this study: prolactin receptor (PRLR)/STAT5, EGFR, and RANK/RANKL. The STAT5 pathway has been shown to mediate PRLR activity and regulate mammary gland development, differentiation, and proliferation [26,34], whereas EGFR is a central growth factor pathway in mammary gland development and a subset of breast cancers [35]. Both PRLR/STAT5 and EGFR pathways are also known targets of progestogen action in the mouse mammary gland [36,37].

RANK/RANKL is the third pathway selectively modified by the combination of CEE + MPA. This pathway has important roles in lymph node development during embryogenesis and is essential for the formation, function, and survival of bone-resorbing osteoclasts [38]. Modulation of the latter mechanism is the basis for the development of the fully human monoclonal antibody to RANKL, denosumab, recently approved for the prevention of skeletal-related events in patients with bone metastases from solid tumors [39]. Analysis of RANK- and RANKL-knockout mice revealed defective mammary alveologenesis [40], which resembled the mammary morphogenic defect observed in PGR-knockout mice [41]. Transcription of RANKL is rapidly induced upon progesterone exposure in mice [30] and co-localized with PGR within “transmitter” ER/PGR-positive luminal mammary epithelial cells [42]. Subsequent studies have shown that RANKL is an essential paracrine mediator of progesterone function in the mouse mammary gland, leading to both mammary epithelial proliferation [43] and the transient expansion and increased regenerative potential of mammary stem cells [44,45] during pregnancy and the estrous cycle. Importantly, these functionalities are not limited to normal mammary morphogenesis and observations in rodent models have now shown that RANKL, via activation of RANK within mammary epithelium, mediates progesterone-dependent mammary tumor formation [28,29].

Currently, it is unclear whether the RANK/RANKL pathway functions similarly in human breast tissue. In the current study, we show that key components of the RANKL pathway are expressed in the normal primate mammary gland and modulated by long-term EPT exposure at clinically relevant doses. Protein expression patterns of RANKL and RANK protein were highly similar to those seen in the mouse [28] and human mammary gland (data not shown) [32]. In each species, RANKL protein is focally expressed in discrete luminal epithelial cells of the ducts and lobules often separated by adjacent RANKL-negative cells. Dual immunolabeling revealed that RANKL protein expression was highly colocalized within PGR-positive luminal epithelial cells in this study, similar to what has been recently described in mice and humans [28,32]. In contrast, RANK protein is segmentally expressed, sporadically in alveoli and often in cells located along the basal aspect of ducts but also in epithelial cells that extend from the basal compartment to the lumen. Similar to observations in mice, RANKL protein levels within the mammary epithelium of macaques were clearly elevated upon exposure to estrogen with a progestin but not estrogen alone. In the CEE + MPA group, we also observed decreased mRNA expression levels of OPG, the negative regulator of RANKL, thereby increasing the ratio of either RANKL/OPG or RANK/OPG. Increased RANKL protein expression was positively associated with increased ductal and alveolar proliferation driven by EPT. RANK protein was colocalized with Ki-67 in a subset of cells, suggesting that RANK-expressing breast cells directly respond to the RANKL signal and comprise at least part of the proliferative component after progestin exposure. Altogether, multiple mechanisms of hormone-dependent control contributing to the net increase in RANKL signal were identified and shown to be positively associated with increased epithelial proliferation (*MKI67*) and density (*KRT19*), suggesting that this pathway may be utilized across mammalian species for progestogen-dependent breast proliferation.

In premenopausal women, ovarian-produced progesterone may also contribute to the established relation between breast cancer risk and number of menstrual cycles or reproductive history [46], potentially via increased breast proliferation [47] and the non-proliferative expansion of normal or transformed mammary stem cells [44,45,48]. Using a candidate gene approach, a recent study identified RANKL, c-Kit, and gene signatures representing MaSC or luminal progenitors as each being associated with younger age at breast cancer diagnosis [49]. Although the relative contribution of RANKL mRNA from normal breast versus tumor tissue was not specified in this analysis of patients with breast cancer, the authors concluded that the strong correlation of these gene sets (including RANKL expression) was independent of breast cancer subtype and instead represented unique biological pathways common to breast cancer in young women and perhaps related to the poor prognosis in these patients. Given the present evidence, the increased mammary mitogenesis observed during the progesterone-dominant luteal phase of the human menstrual cycle [47] could involve an operative role of RANKL. Recent gene expression analysis of fine-needle aspirates of human breast tissue demonstrating significant upregulation of RANKL mRNA during the luteal phase [50] is consistent with this notion. In fact, a recent publication [32] has demonstrated that RANKL levels in the human breast are correlated with serum progesterone levels. Furthermore, RANKL was not only sufficient to induce human breast cell proliferation but was also required for progesterone-induced breast cell proliferation. These data, with observations presented in this primate study, suggest that the increased RANKL signal in human breast tissue is a consequence of progestogen exposure in postmenopausal women or luteal phase ovarian progesterone in premenopausal women. Moreover, this increased RANKL may be correlated with the proliferative status and overall density of the mammary epithelium and contribute to hormone-dependent breast tumor formation.

The three signaling pathways identified here as being selectively increased by EPT all exhibit signaling cross-talk that may be functionally important in breast cancer. Prior studies have shown that the induction of RANKL by MPA requires expression of PRLR and that prolactin signaling is necessary for nuclear translocation of STAT5A after EPT [29,51]. These findings also indicate that the interferon-gamma responsive elements identified within the RANKL promoter are essential for activation of the JAK2/STAT5A response [51] and potentially important for progestogen-dependent increases. Other studies have shown that nuclear phosphorylated STAT5A is co-localized with PGR and RANKL in cells after EPT [26], further suggesting that progestogen-dependent increases in RANKL transcription may be governed at the RANKL promoter, at least in part, by a complex of PGR and STAT5A, similar to that observed with the β-casein promoter [52]. Finally, EGFR ligands have been shown to strongly decrease OPG expression in an EGFR-dependent manner [27] and activate STAT5A in mammary tissue [53]. Collectively, these data support a model in which progestogen activity in breast tissue may increase RANKL protein expression either directly, or indirectly, via PRLR/STAT5 signaling, whereas OPG protein expression may be decreased via EGFR signaling. Future studies are warranted to determine if multifactorial convergences of the PRLR/STAT5, EGFR, and RANK/RANKL pathways may contribute to breast cancer risk.

Unlike EPT, tibolone did not show a clear induction of mRNA markers in breast tissue related to growth factor signaling. This finding is consistent with the lack of increased mammary epithelial Ki-67 labeling reported previously [21] and the lack of increased breast cancer risk among older postmenopausal women receiving tibolone in the LIFT clinical trial [12]. In contrast, tibolone treatment in the LIBERATE clinical trial was associated with a 40% increased risk of recurrence among breast cancer patients with ER-positive tumors and vasomotor symptoms [15]. Sub-group analysis among women receiving adjuvant endocrine therapy indicated that tibolone interference was greater for aromatase inhibitors (which decrease systemic and local estrogen) than for tamoxifen (which blocks ERs). The authors of the study suggest that these findings point to potential ER agonist effects of tibolone on occult, estrogen-sensitive metastases [15]. Results from the current study provide limited support for this idea, showing modest estrogenic effects of tibolone on some gene markers of ER activity but no clear progestogenic effects or activity related to specific growth factor pathways.

## Conclusions

Minimizing breast cancer risk by mitigating potential adverse effects of hormonal agents is a central challenge in women’s health. Results of this study expand the current understanding of transcriptional patterns and signaling pathways underlying HT effects in mammalian breast tissue. Findings presented here identify PRLR/STAT5, EGFR, and RANK/RANKL as molecular pathways that may be relevant to increased breast tissue proliferation, mammographic density, and breast cancer risk in postmenopausal women taking EPT. These pathways are potential targets for assessing and preventing progestogen-associated risk, and this information should help inform clinical strategies to better prevent hormone-associated breast cancer and recurrence.

## Abbreviations

ACTB: Beta-actin; ANOVA: Analysis of variance; AREG: Amphiregulin; CEE: Conjugated equine estrogens; EGFR: Epidermal growth factor receptor; EPT: Estrogen + progestin therapy; ER: Estrogen receptor; ESR1: Estrogen receptor-alpha; ESR2: ER-beta; ET: Estrogen-alone therapy; FC: Fold change; GAPDH: Glyceraldehyde-3-phosphate dehydrogenase; GREB1: Growth regulation by estrogen in breast cancer 1; HSD17B1: 17-beta hydroxysteroid dehydrogenase type 1; HSD17B2: 17-beta hydroxysteroid dehydrogenase type 2; HSD3B: 3-beta-hydroxysteroid dehydrogenase/delta-5-delta-4 isomerase; HT: Hormone therapy; IGFBP1: Insulin-like growth factor binding protein 1; IHC: Immunohistochemical; KRT19: Keratin 19; MKI67: Antigen identified by monoclonal antibody Ki-67; MPA: Medroxyprogesterone acetate; OPG: Osteoprotegerin; PCA: Principal components analysis; PGR: Progesterone receptor; PRLR: Prolactin receptor; qPCR: Quantitative real-time polymerase chain reaction; RANK: Receptor activator of nuclear factor kappa B; RANKL: Receptor activator of nuclear factor kappa B ligand; SA-HRP: Streptavidin-horseradish peroxidase; STAT5: Signal transducer and activator of transcription 5; TFF1: Trefoil factor 1; TGF: Transforming growth factor; Tib: Tibolone; WHI: Women’s Health Initiative.

## Competing interests

CW has received research funding from Amgen Inc and Pfizer; DB, AJ, KR, LH, and WD are employees and have received stock from Amgen Inc; JC has consulted and received research funding from Amgen Inc; TR and HB have no disclosures.

## Authors’ contributions

Conception and design: CW, WD, and DB. Development of methodology: CW, WD, DB, HB, LH, KR, TR. Acquisition of data: CW, WD, LH, DB, and JC. Analysis and interpretation of data: CW, AJ, WD, DB, and JC. Writing, review, and/or revision of the manuscript: CW, AJ, WD, DB, TR, and JC. All authors read and approved the manuscript.

## Supplementary Material

Additional file 1: Table S1 Primer/probe sets for target genes evaluated by quantitative PCR. Click here for file

Additional file 2: Figure S1 Regression analysis for expression of the proliferation marker *MKI67* and markers of STAT5, epidermal growth factor receptor (EGFR), and estrogen receptor (ER) signaling pathways. (a) *MKI67* mRNA versus STAT5 markers; (b) *MKI67* mRNA versus EGFR markers; and (c) *MKI67* mRNA versus ER markers. Across all groups, the strongest positive correlations were observed between *MKI67* versus *STAT5A, PRLR,* amphiregulin (*AREG), and* transforming growth factor-alpha *(TGFA)* (*P* < 0.0001 for all). Click here for file

Additional file 3: Figure S2 Regression analysis for *MKI67* versus *RANKL* and *RANK*. (a) *RANKL* mRNA versus *MKI67*. (b) *RANK* mRNA versus *MKI67*. Significant positive correlations were observed between *RANK* versus *MKI67* (R = 0.48, *P* < 0.0001). Click here for file

Additional file 4: Figure S3 Regression analysis for *STAT5A* or a marker for breast density (*KRT19*) versus *RANKL*/*OPG* or *RANK*/*OPG* mRNA ratios. (a) *RANKL*/*OPG* mRNA ratio versus *STAT5A*. (b) *RANK*/*OPG* mRNA ratio versus *STAT5A*. (c) *RANKL*/*OPG* mRNA ratio versus *KRT19*. (d) *RANK*/*OPG* mRNA ratio versus *KRT19*. Significant positive correlations were observed between *RANK*/*OPG* versus *STAT5A* (R = 0.69, *P* < 0.0001) and *RANK*/*OPG* versus *KRT19* (R = 0.55, *P* < 0.0001). *RANKL*/*OPG* versus *STAT5A or KRT19* show modest positive correlations (R = 0.35, *P* = 0.0008 and R = 0.29, *P* = 0.006, respectively). Click here for file

Additional file 5: Figure S4 RANKL is localized in PGR-positive mammary luminal epithelial cells. Representative examples of immunohistochemical co-labeling of RANKL and PGR in breast tissue from conjugated equine estrogens plus medroxyprogesterone acetate (CEE + MPA) treated monkeys. RANKL is red and PGR is brown. (a) RANKL and PGR labeling in breast duct (40X); (b) RANKL and PGR labeling in lobuloalveolar structure (60X). RANKL and PGR protein is clearly evident exclusively within the luminal epithelial cells of the lobuloalveolar and ductal epithelium. Cytoplasmic and membrane RANKL expression is localized in PGR expressing cells. Click here for file

Additional file 6: Figure S5 Correlation of either RANKL or RANK protein expression with corresponding mRNA expression. (a) RANKL protein immunohistochemical (IHC) score versus *RANKL* mRNA. A significant positive correlation was observed in the conjugated equine estrogens plus medroxyprogesterone acetate (CEE + MPA) group (R = 0.62, *P* = 0.0004) only. The majority of animals within the control and CEE groups (100% and 88%, respectively) did not express RANKL by IHC, precluding correlation analysis between protein and mRNA expression in these groups. (b) RANK protein IHC score versus *RANK* mRNA. There were no significant correlations in any group. Click here for file

Additional file 7: Figure S6 Correlation of RANK protein expression with Ki-67 protein expression. RANK composite immunohistochemical (IHC) score versus log_10_ Ki-67 positive cells (%) in (a) lobuloalveolar or (b) ductal cells. No correlation between RANK IHC score and Ki-67 positive cells (%) was observed. Click here for file

Additional file 8: Figure S7 Dual labeling of RANK protein expression and Ki-67 in breast epithelium from conjugated equine estrogens plus medroxyprogesterone acetate (CEE + MPA) treated monkeys. (a to d) Representative examples of immunohistochemical (IHC) co-labeling of RANK and Ki-67 in breast tissue from CEE + MPA-treated monkeys. RANK is red and Ki-67 is brown. Nuclear staining of Ki-67 was observed in a subset of cells with cytoplasmic and membrane expression of RANK. Low magnification views (a, 10X; b, 40X) of breast tissue demonstrate segmental foci of breast epithelium stained positively for RANK that were also frequently positive for Ki-67 (as circumscribed by dashed lines). Conversely, RANK-negative regions of the same breast tissue often had few or no Ki-67 labeled cells. (c and d) Higher magnification views (60X) show clear examples of individual cells positive for both RANK and Ki-67 in (c) ducts and (d) lobuloalveolar structures. Individual ductal cells positive for both Ki-67 and RANK are indicated by arrows. In the example of staining in lobuloalveolar tissue, the majority of this particular segment stains positively for RANK (similar to that shown in Figure 6b and Additional file 8: Figure S7a, b) with Ki-67 staining in a subset of these RANK-positive cells. Click here for file
