# Supplementary material for: Progestin effects on cell proliferation pathways in the postmenopausal mammary gland
Source: Breast Cancer Res. 2013 Aug 12;15(4):R62. doi: 10.1186/bcr3456 (PMC3978455; doi:10.1186/bcr3456)
Supplement: Additional file 1: Table S1 — Primer/probe sets for target genes evaluated by quantitative PCR. [file bcr3456-S1.doc]

| **Additional** **Table 1 Primer/probe sets for target genes evaluated by qPCR** | | | |
| --- | --- | --- | --- |
| *Gene ID* | *GenBank #* | *Species* | *ABI assay ID* |
| *ACTB* | DQ464112 | *Mf* | (custom) |
| *AREG* | NM_001657 | *Hs* | Hs00950668_m1 |
| *BMP2* | XM_001115987 | *Rh* | Rh02912831_m1 |
| *CYP19* | DQ529980 | *Mf* | (custom) |
| *DCN* | NM_133503 | *Hs* | Hs00754870_s1 |
| *EGF* | NM_001963 | *Hs* | Hs00153181_m1 |
| *ESR1* | DQ469336 | *Mf* | (custom) |
| *ESR2* | HQ702565 | *Mf* | (custom) |
| *GAPDH* | DQ464111 | *Mf* | (custom) |
| *GREB1* | NM_033090 | *Mm* | Rh02866842_m1 |
| *HSD17B1* | DQ529983 | *Mf* | (custom) |
| *HSD17B2* | DQ529984 | *Mf* | (custom) |
| *KRT19* | NM_002276 | *Hs* | Hs00761767_s1 |
| *MKI67* | NM_002417 | *Hs* | Hs00606991_m1 |
| *OPG^1^* | NM_002546 | *Hs* | Hs00171068_m1 |
| *PGR* | NM_000926 | *Hs* | Hs00172183_m1 |
| *PRLR* | NM_000949 | *Hs* | Hs00168739_m1 |
| *RANK^2^* | NM_003839 | *Hs* | Hs00187189_m1 |
| *RANKL^3^* | NM_003701 | *Hs* | Hs00243522_m1 |
| *STAT5A* | NM_003152 | *Mm* | Rh02844604_m1 |
| *STAT5B* | NM_012448 | *Hs* | Hs00560035_m1 |
| *STS* | DQ529981 | *Mf* | (custom) |
| *SULT1E* | DQ529982 | *Mf* | (custom) |
| *TFF1* | DQ464113 | *Mf* | (custom) |
| *TGFA* | NM_003236 | *Mm* | Rh00608187_m1 |
| *TGFB2* | NM_003238 | *Hs* | Hs01548878_m1 |
| *Hs*, *Homo sapiens*; *Mf*, *Macaca fascicularis* (cynomolgus macaque); *Mm*, *Macaca mullata* (rhesus macaque); *^1^OPG = TNFRSF11B;  ^2^RANK = TNFRSF11A; ^3^RANKL = TNFSF11* | | | |
