# Supplementary figures and images for: Progestin effects on cell proliferation pathways in the postmenopausal mammary gland
Source: Breast Cancer Res. 2013 Aug 12;15(4):R62. doi: 10.1186/bcr3456 (PMC3978455; doi:10.1186/bcr3456)

**A**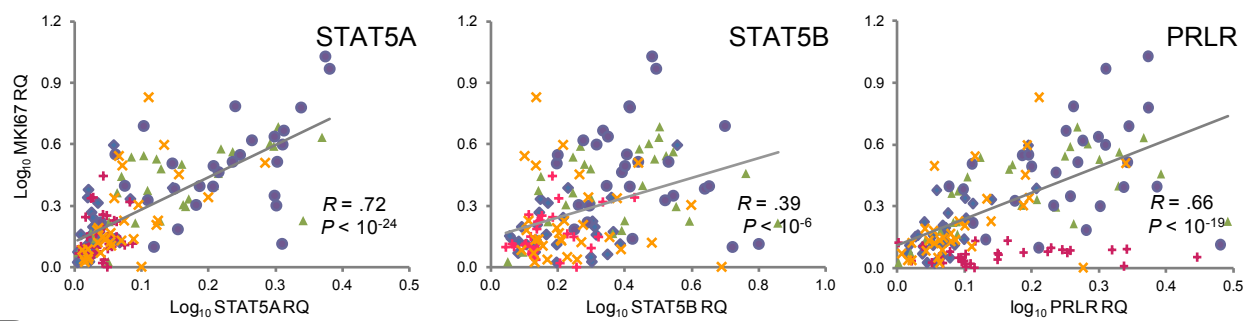**B**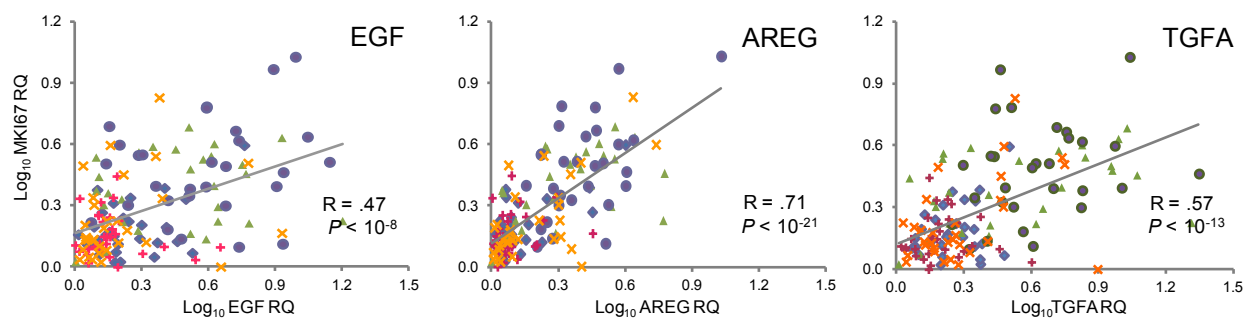**C**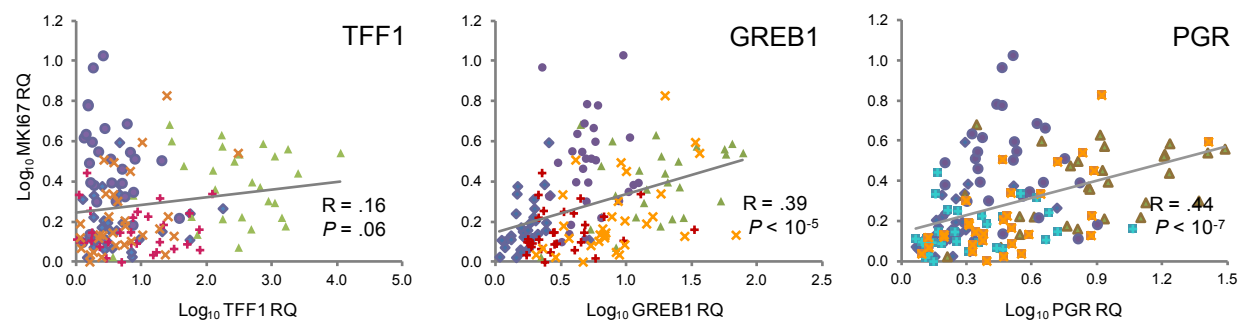

◆ Con    ▲ CEE    ● CEE+MPA    + Lo Tib    × Hi Tib

Supplement: Additional file 2: Figure S1 — Regression analysis for expression of the proliferation marker MKI67 and markers of STAT5, epidermal growth factor receptor (EGFR), and estrogen receptor (ER) signaling pathways. (a) MKI67 mRNA versus STAT5 markers; (b) MKI67 mRNA versus EGFR markers; and (c) MKI67 mRNA versus ER markers. Across all groups, the strongest positive correlations were observed between MKI67 versus STAT5A, PRLR, amphiregulin (AREG), and transforming growth factor-alpha (TGFA) (P < 0.0001 for all). [file bcr3456-S2.pdf]

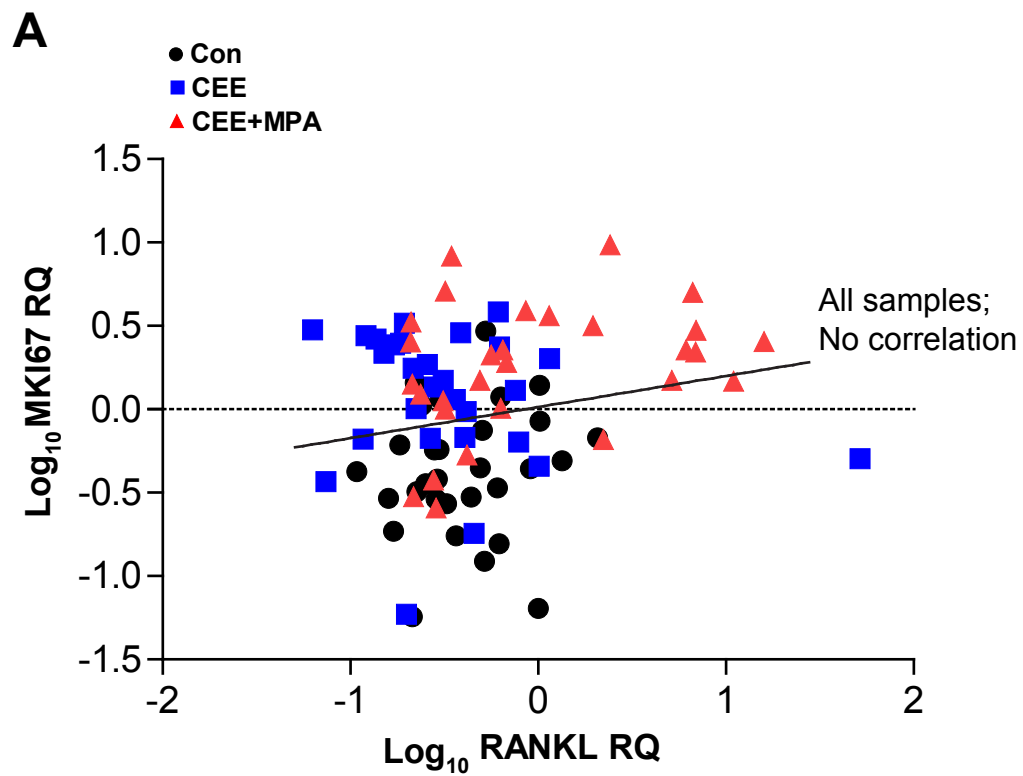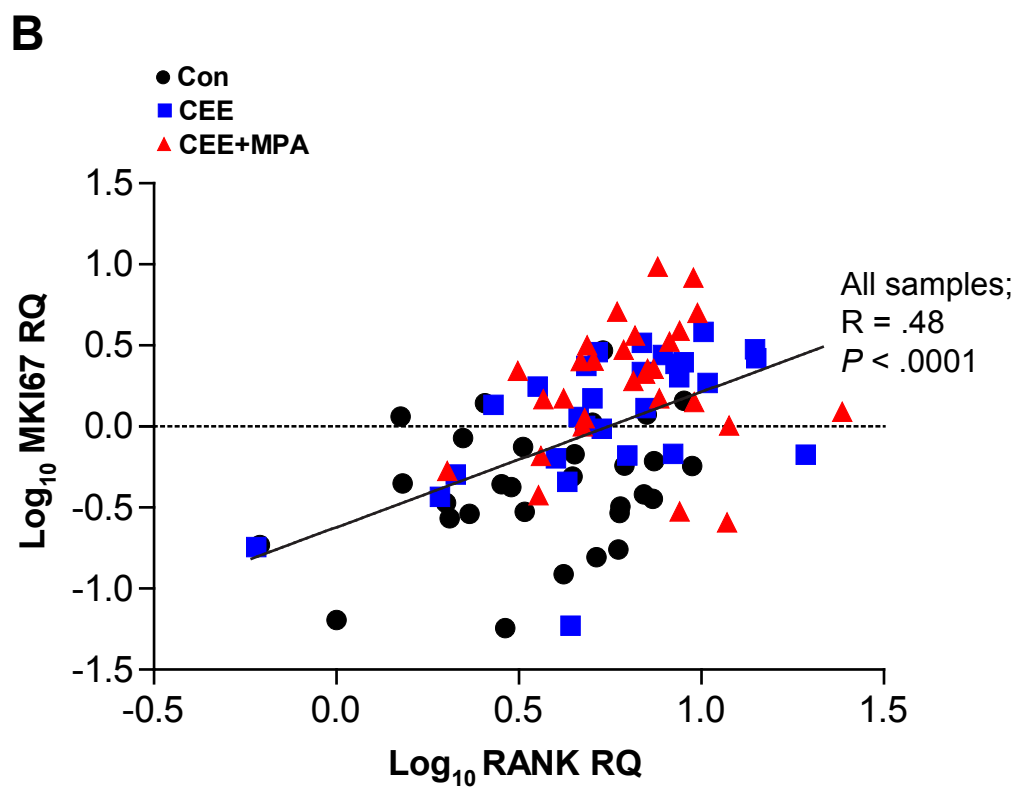

Supplement: Additional file 3: Figure S2 — Regression analysis for MKI67 versus RANKL and RANK. (a) RANKL mRNA versus MKI67. (b) RANK mRNA versus MKI67. Significant positive correlations were observed between RANK versus MKI67 (R = 0.48, P < 0.0001). [file bcr3456-S3.pdf]

**A**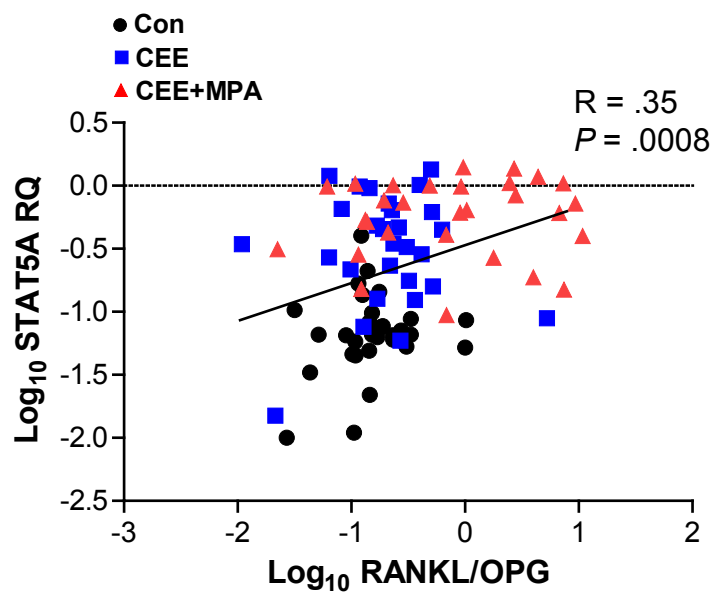**B**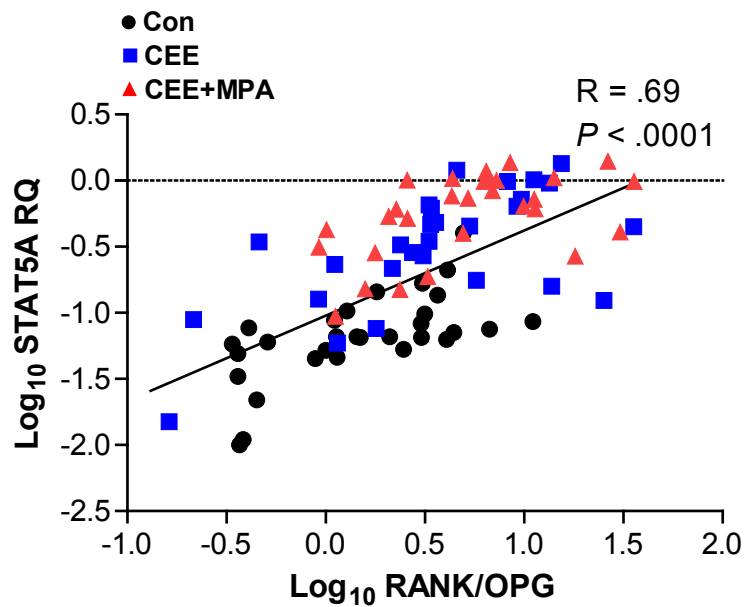**C**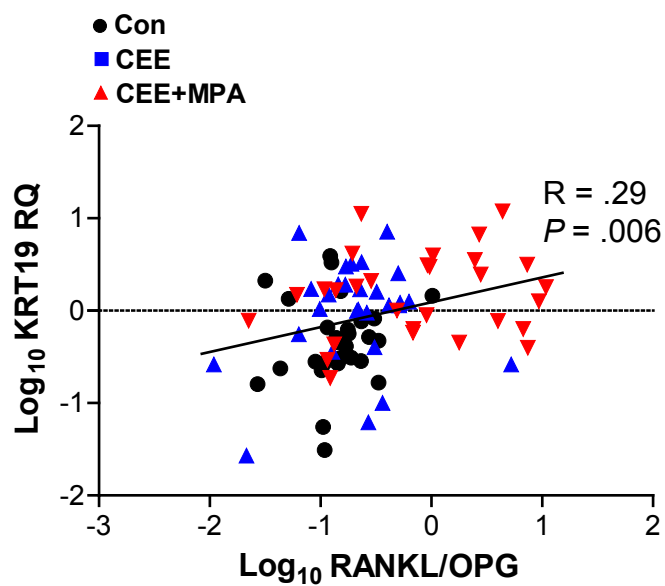**D**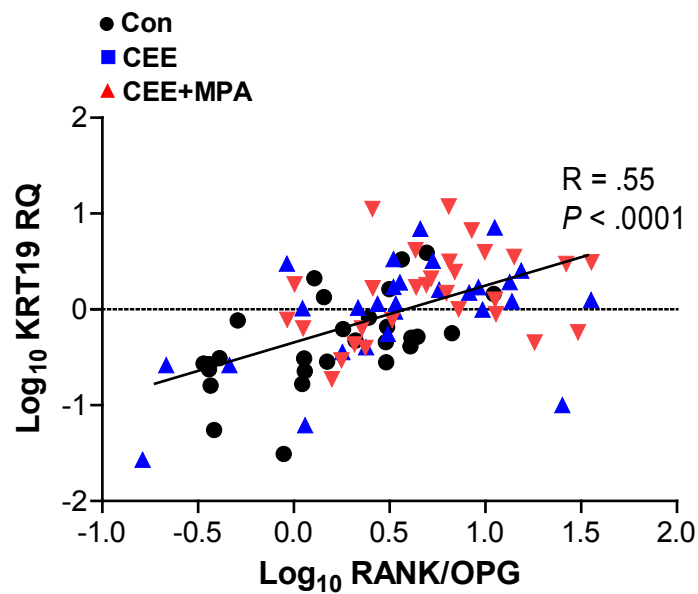

Supplement: Additional file 4: Figure S3 — Regression analysis for STAT5A or a marker for breast density (KRT19) versus RANKL/OPG or RANK/OPG mRNA ratios. (a) RANKL/OPG mRNA ratio versus STAT5A. (b) RANK/OPG mRNA ratio versus STAT5A. (c) RANKL/OPG mRNA ratio versus KRT19. (d) RANK/OPG mRNA ratio versus KRT19. Significant positive correlations were observed between RANK/OPG versus STAT5A (R = 0.69, P < 0.0001) and RANK/OPG versus KRT19 (R = 0.55, P < 0.0001). RANKL/OPG versus STAT5A or KRT19 show modest positive correlations (R = 0.35, P = 0.0008 and R = 0.29, P = 0.006, respectively). [file bcr3456-S4.pdf]

**A**

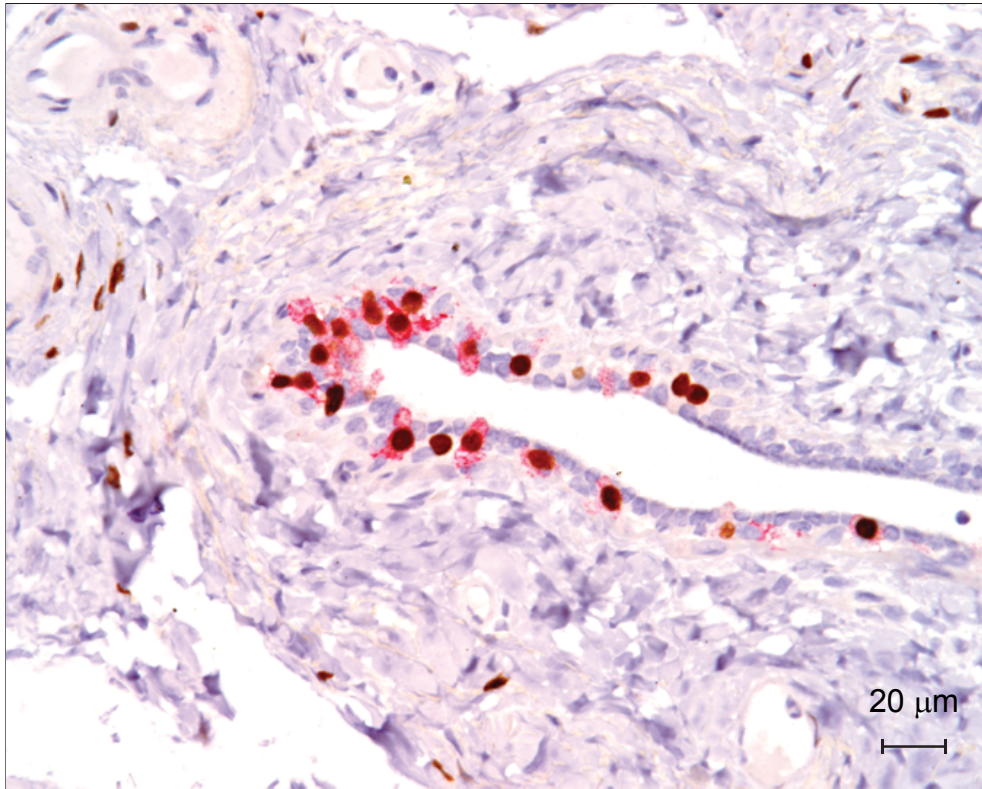

**B**

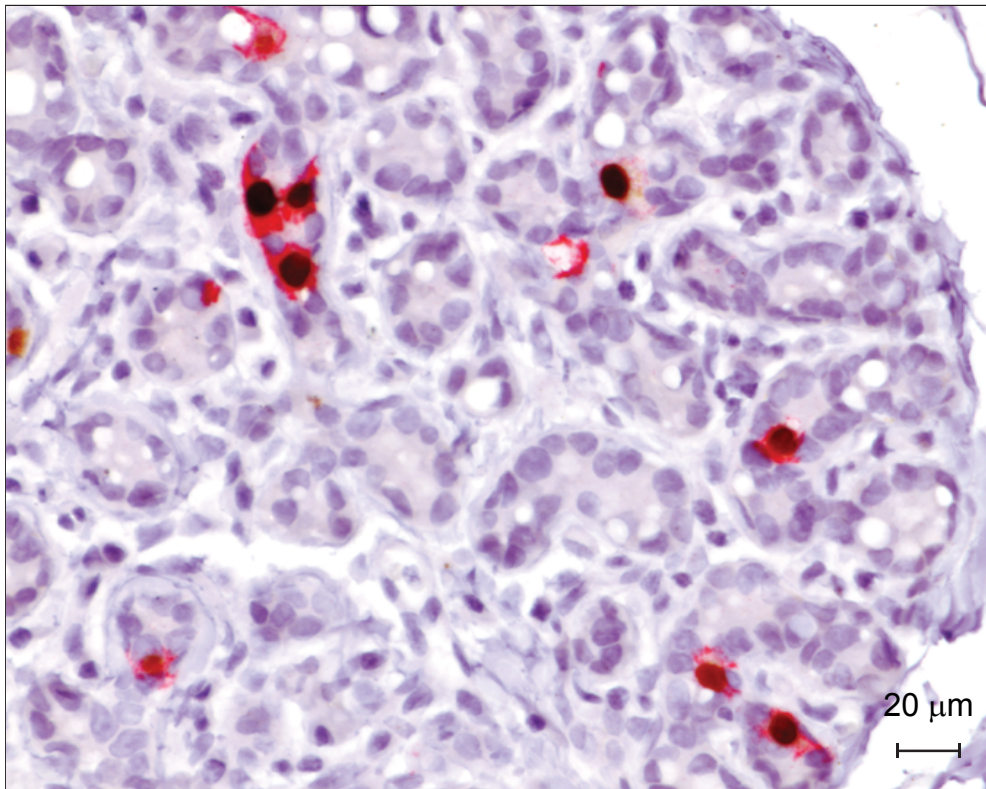

Additional Figure 4

Supplement: Additional file 5: Figure S4 — RANKL is localized in PGR-positive mammary luminal epithelial cells. Representative examples of immunohistochemical co-labeling of RANKL and PGR in breast tissue from conjugated equine estrogens plus medroxyprogesterone acetate (CEE + MPA) treated monkeys. RANKL is red and PGR is brown. (a) RANKL and PGR labeling in breast duct (40X); (b) RANKL and PGR labeling in lobuloalveolar structure (60X). RANKL and PGR protein is clearly evident exclusively within the luminal epithelial cells of the lobuloalveolar and ductal epithelium. Cytoplasmic and membrane RANKL expression is localized in PGR expressing cells. [file bcr3456-S5.pdf]

**A**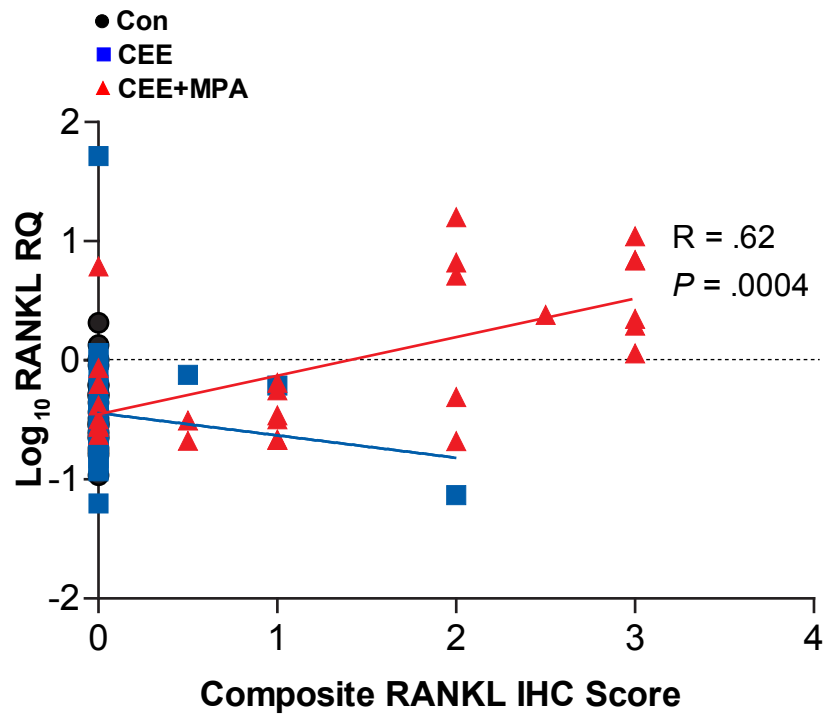**B**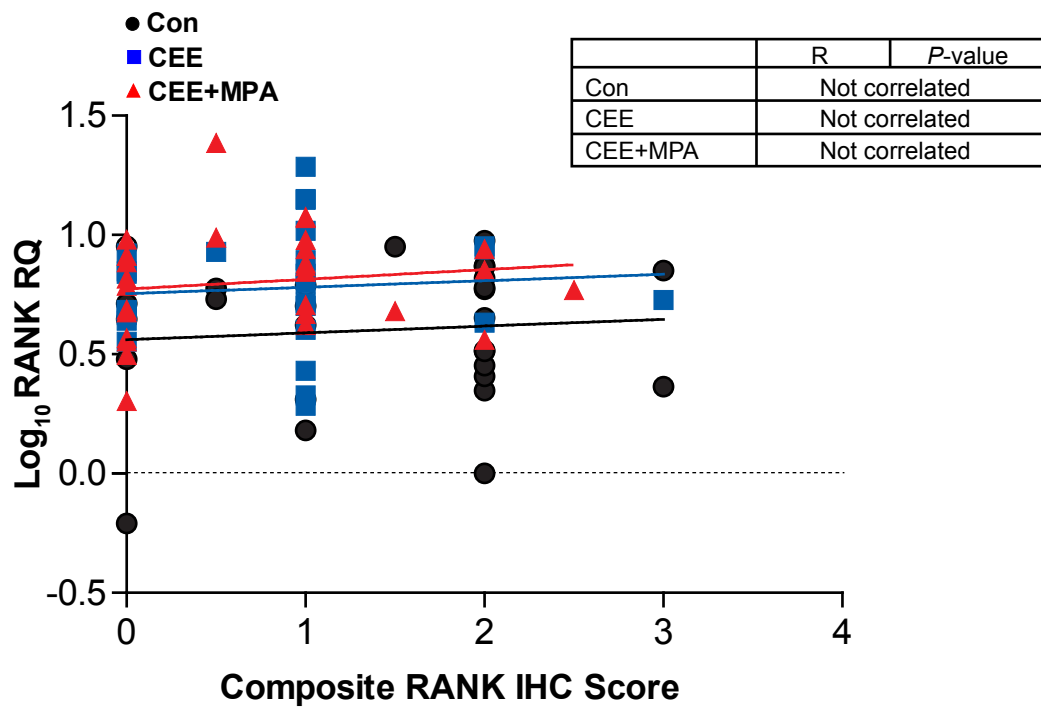

Supplement: Additional file 6: Figure S5 — Correlation of either RANKL or RANK protein expression with corresponding mRNA expression. (a) RANKL protein immunohistochemical (IHC) score versus RANKL mRNA. A significant positive correlation was observed in the conjugated equine estrogens plus medroxyprogesterone acetate (CEE + MPA) group (R = 0.62, P = 0.0004) only. The majority of animals within the control and CEE groups (100% and 88%, respectively) did not express RANKL by IHC, precluding correlation analysis between protein and mRNA expression in these groups. (b) RANK protein IHC score versus RANK mRNA. There were no significant correlations in any group. [file bcr3456-S6.pdf]

**A**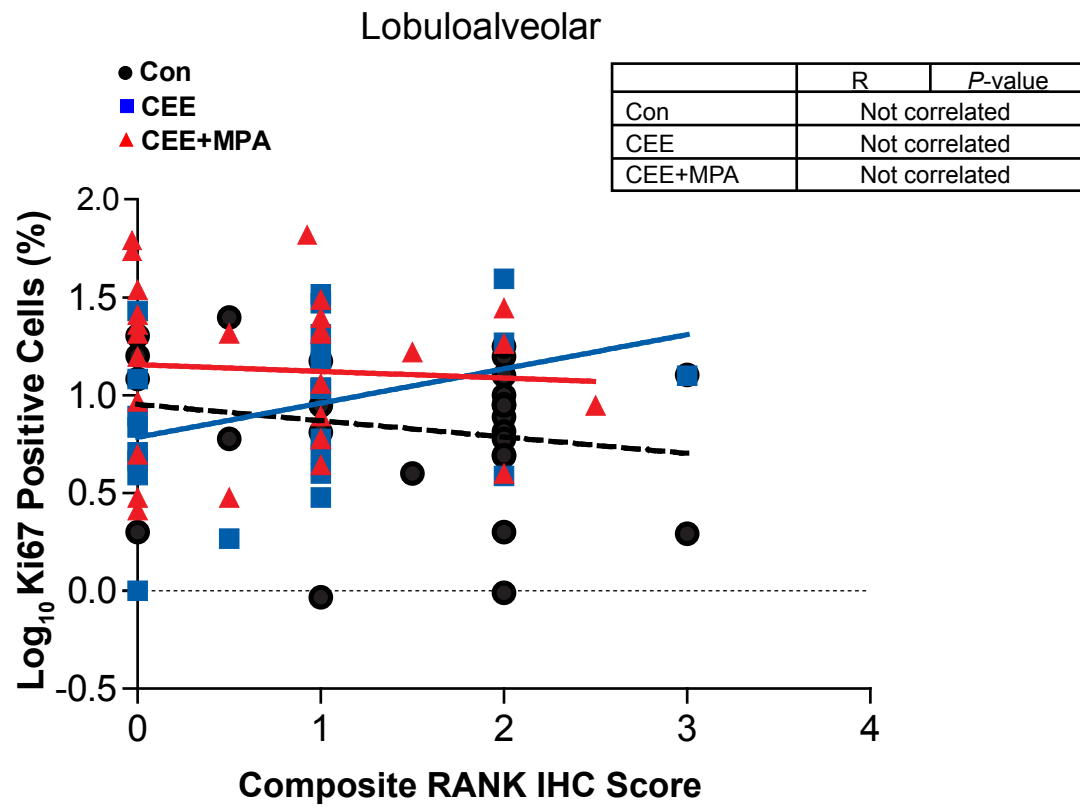**B**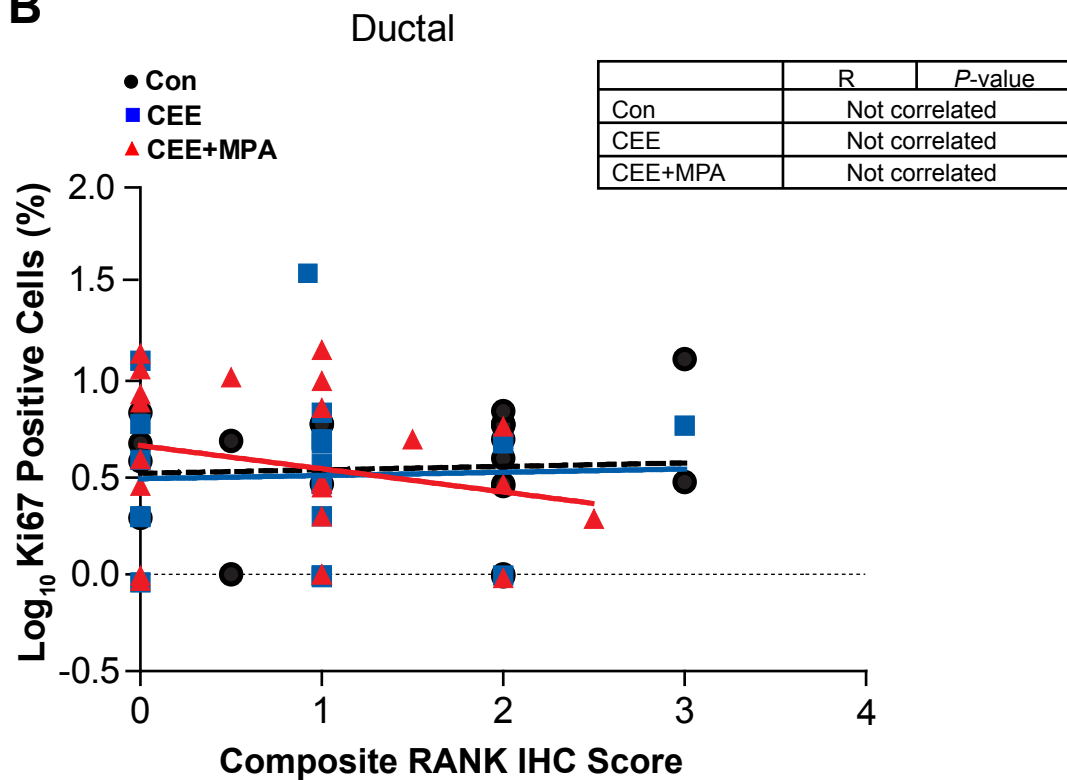

Supplement: Additional file 7: Figure S6 — Correlation of RANK protein expression with Ki-67 protein expression. RANK composite immunohistochemical (IHC) score versus log10 Ki-67 positive cells (%) in (a) lobuloalveolar or (b) ductal cells. No correlation between RANK IHC score and Ki-67 positive cells (%) was observed. [file bcr3456-S7.pdf]

**A**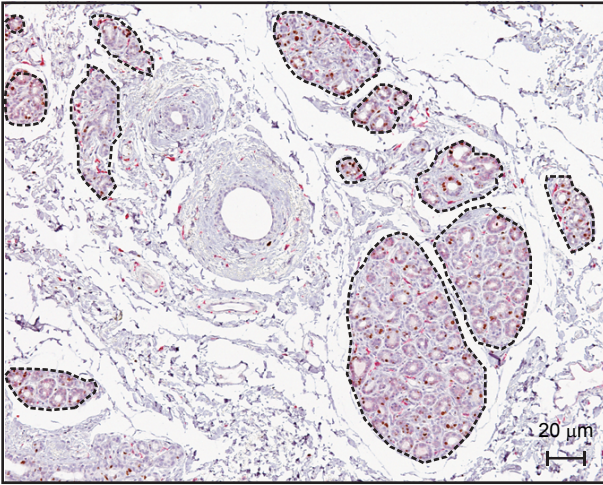**B**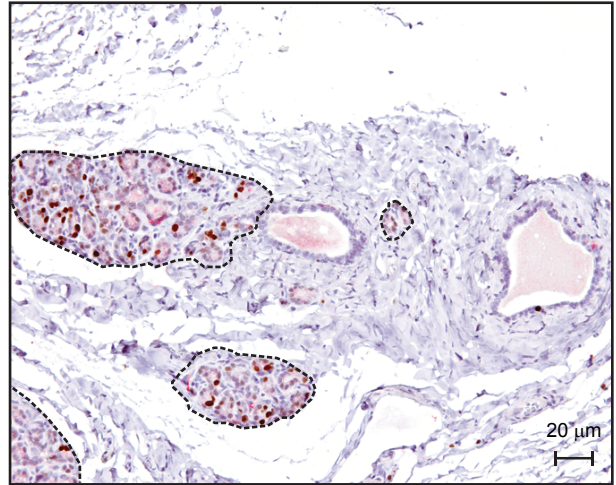**C**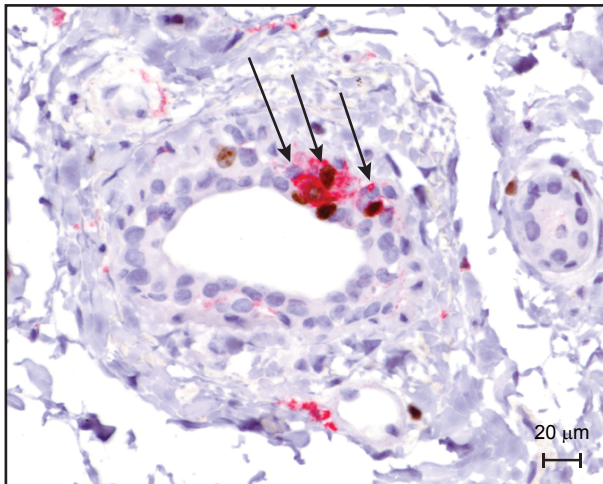**D**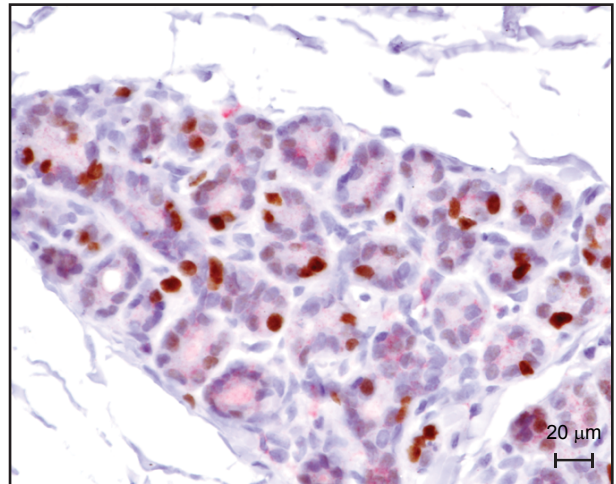

Supplement: Additional file 8: Figure S7 — Dual labeling of RANK protein expression and Ki-67 in breast epithelium from conjugated equine estrogens plus medroxyprogesterone acetate (CEE + MPA) treated monkeys. (a to d) Representative examples of immunohistochemical (IHC) co-labeling of RANK and Ki-67 in breast tissue from CEE + MPA-treated monkeys. RANK is red and Ki-67 is brown. Nuclear staining of Ki-67 was observed in a subset of cells with cytoplasmic and membrane expression of RANK. Low magnification views (a, 10X; b, 40X) of breast tissue demonstrate segmental foci of breast epithelium stained positively for RANK that were also frequently positive for Ki-67 (as circumscribed by dashed lines). Conversely, RANK-negative regions of the same breast tissue often had few or no Ki-67 labeled cells. (c and d) Higher magnification views (60X) show clear examples of individual cells positive for both RANK and Ki-67 in (c) ducts and (d) lobuloalveolar structures. Individual ductal cells positive for both Ki-67 and RANK are indicated by arrows. In the example of staining in lobuloalveolar tissue, the majority of this particular segment stains positively for RANK (similar to that shown in Figure 6b and Additional file 8: Figure S7a, b) with Ki-67 staining in a subset of these RANK-positive cells. [file bcr3456-S8.pdf]
